# Development of a Mass Sensitive Quartz Crystal Microbalance (QCM)-Based DNA Biosensor Using a 50 MHz Electronic Oscillator Circuit

**DOI:** 10.3390/s110807656

**Published:** 2011-08-03

**Authors:** Gonzalo García-Martinez, Enrique Alonso Bustabad, Hubert Perrot, Claude Gabrielli, Bogdan Bucur, Mathieu Lazerges, Daniel Rose, Loreto Rodriguez-Pardo, Jose Fariña, Chantal Compère, Antonio Arnau Vives

**Affiliations:** 1 Dpto. Tecnología Electrónica, Universidad de Vigo, Campus Lagoas Marcosende, Vigo 36310, Spain; E-Mails: skevere@gmail.com (G.G.-M.); torpedix@hotmail.com (E.A.B.); lpardo@uvigo.es (L.R.-P.); jfarina@uvigo.es (J.F.); 2 LISE (UPR15), CNRS/4, Place Jussieu, cp 133, Paris cedex 05 75252, France; E-Mails: claude.gabrielli@upmc.fr (C.G.); bucur.bogdan@upmc.fr (B.B.); lazerges@ccr.jussieu.fr (M.L.); daniel.rose@upmc.fr (D.R.); 3 Université P. et M. Curie/4, place Jussieu, cp 133, Paris cedex 05 75252, France; 4 IFREMER, Service Interfaces et Capteurs, Centre de Brest, BP 70, Plouzané 29280, France; E-Mail: chantal.compere@ifremer.fr; 5 Universidad Politécnica de Valencia, ETSI de Telecomunicación, Camino de Vera s/n, Valencia 46022, Spain; E-Mail: aarnau@eln.upv.es

**Keywords:** DNA sequence detection, quartz crystal microbalance, electronic oscillator, sensitivity, frequency noise, resolution

## Abstract

This work deals with the design of a high sensitivity DNA sequence detector using a 50 MHz quartz crystal microbalance (QCM) electronic oscillator circuit. The oscillator circuitry is based on Miller topology, which is able to work in damping media. Calibration and experimental study of frequency noise are carried out, finding that the designed sensor has a resolution of 7.1 ng/cm^2^ in dynamic conditions (with circulation of liquid). Then the oscillator is proved as DNA biosensor. Results show that the system is able to detect the presence of complementary target DNAs in a solution with high selectivity and sensitivity. DNA target concentrations higher of 50 ng/mL can be detected.

## 1. Introduction

Biosensors are small devices which utilize biological reactions for detecting target analytes [1–3]. Such devices intimately couple a biological recognition element (interacting with the target analyte) with a physical transducer that translates the bio-recognition event into a useful electrical signal. Common transducing elements, including optical, electrochemical or mass-sensitive devices, generate light, current or frequency signals, respectively. There are two types of biosensors, depending on the nature of the recognition event. Bio-affinity devices rely on the selective binding of the target analyte to a surface-confined ligand partner (e.g., antibody, oligonucleotide) [4]. In contrast, in bio-catalytic devices, an immobilized enzyme is used for recognizing the target substrate.

Specific DNA sequence detection is a major issue in life science. An important advance in this field was done during the last two decades with the design of DNA biosensors. They are more efficient by comparison to DNA hybridization tests performed on membranes that are less sensitive, less selective, time consuming and not time resolved. DNA biosensors are now intensely developed for diagnostic applications, environmental monitoring and food controls. DNA detection biosensors are based in the hybridization process of combining complementary, single-stranded DNA into a single molecule.

The quartz crystal microbalance (QCM) oscillator circuits are useful to design DNA-biosensors [5]. A QCM sensor typically consists of an oscillator circuit containing a thin AT-cut quartz disc with circular electrodes on both sides of the quartz. Due to the piezoelectric properties of the quartz material, an alternating voltage between these electrodes leads to a mechanical oscillations of the crystal. These oscillations are generally very stable due to the high quality of the quartz (high Q factor). If a mass is adsorbed or placed onto the quartz crystal surface, the frequency of oscillation changes in proportion to the amount of mass. Therefore, these devices can be used as high sensitivity microbalances intended to measure mass changes in the nanogram range by coating the crystal with a material which is selective towards the species of interest. The quartz crystal acts as a signal transducer, converting mass changes due to the hybridization process into frequency changes. One of the main advantages of this device is the ability to control a QCM’s selectivity by applying different coatings, which makes this sensor type extremely versatile.

**Figure 1.**
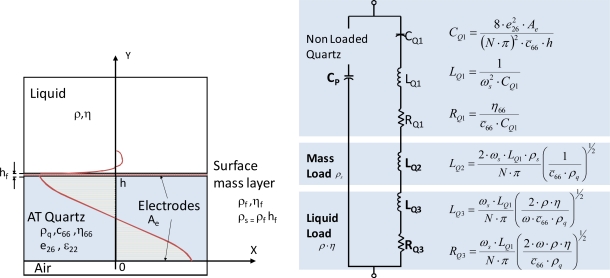
Electromechanical model of a piezoelectric resonator for microgravimetrical applications in liquid; Cross-section of a loaded resonator and BVD equivalent circuit modified by Martin and Granstaff.

The design of crystal controlled oscillators used as QCM sensors in fluids is a difficult task due to the wide dynamic values of the resonator resistance that they should support during their operations [6]. The piezoelectric quartz experiences a strong reduction of its quality factor due to the increase of the losses (*R*_Q_) caused by the liquid. Figure 1 shows the BVD equivalent circuit of a piezoelectric resonator modified by Martin and Granstaff [7] for a quartz crystal loaded by the mass of a material layer and a liquid. The standard oscillator designs, as Pierce or Colpitts, does not work well since, although they provide a great stability in frequency and a low phase noise, their gain and phase are very sensitive to the losses of the resonator [8]. A good design of a sensor oscillator for liquid media will maintain the necessary loop gain and phase for the oscillation (Barhausen condition) in a wide margin of values of the loss resistances of the quartz.

This work deals with the design and implementation of a high frequency QCM electronic oscillator circuit for its use as high sensitivity DNA biosensor. The QCM oscillator sensor is able to detect the presence of complementary DNAs in a solution that match the sequence on a given strand in function of the changes in the output frequency of the oscillator. The design is adapted so that the Barkhausen conditions are satisfied even when the quartz is immersed in liquid media. An experimental characterization of the frequency stability of the oscillator is carried out, with object of determining the resolution of the sensor. The behavior of the oscillator as DNA biosensor is proven, by monitoring its frequency during the process of immobilization of probe DNA on the gold-covered quartz surface of the QCM oscillator and during the hybridization of complementary target DNA present in a solution. Finally, a calibration of the DNA biosensor with buffer solutions of different target DNA concentrations is carried out and the minimum concentration of DNA detectable is determined.

## 2. Experimental Section

A home-made quartz crystal electronic oscillator circuit was designed to drive the quartz at its resonance frequency and use it as QCM sensor in liquid media. Miller oscillator topology was selected, and a working frequency of 50 MHz was chosen in order to have a high sensitivity QCM sensor system [9].

**Figure 2.**
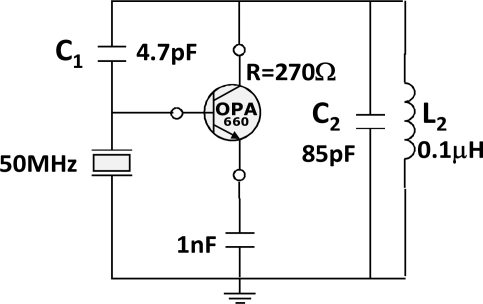
Schematic of the Miller oscillator circuit.

The Miller topology is a high-frequency stable topology that allows designing sensors of high resolution [10–12]. This topology, in spite of not being the most adequate topology for obtaining the best frequency stability [13], experimentally showed a good capacity to work under strong damping [14,15]. Miller oscillators allow solving the problems that have the standard oscillators to work in liquid, as Colpitts or Clapp, thanks to its ability of supporting a wide range of values of the resonator resistance due to the damping. Once decided the configuration, the design and simulation of the circuit was done with the help of PSpice. To model the quartz resonator, the experimental values of the parameters of the equivalent electric circuit in distilled water were used. They are summarized in Table 1. The Burr-Brown OTA660 transconductance amplifier was used as the active device [16]. To determine the values of the components, the design considerations for this topology in [11,12] were realized. The OTA was polarized using a resistance of 270 Ω to have a high gain. In Figure 2, a simplified scheme of the designed oscillator is shown.

Once the oscillator circuit was designed and simulated with PSpice, it was implemented on a printed circuit board (PCB). A temperature sensor was also incorporated to externally control the temperature of the circuit by means of a WATLOW heater monitor with stability better than 0.1 °C. Quartz crystal resonator was connected by a silver conducting paste, through wires, to a BNC adaptator, which permits the connection of the quartz to the oscillator circuitry. An experimental cell was developed: the crystal was mounted between two O-ring seals inserted in a plexiglass cell [17].

The electronic oscillator circuit was experimentally characterized in the measurement environment. The output frequency of the oscillator was connected to a Fluke PM6685 frequency counter controlled by a lab-made software program that allows storing the frequency samples. The temperature of the electronic circuit was controlled by a Watlow regulator. Experiments were made with the plexiglass cell (and therefore the quartz and its environment) included in a BMT Climacell climatic cell which allows maintaining constant the ambient temperature and humidity. A micropump (Pharmacia, P1) was used to provide a constant flow of liquid circulating over the surface of the crystal. The flow rate was chosen low (50 μL/min) to minimize noise in the quartz.

In order to characterize the designed system, a study of the frequency stability of the oscillator was carried out by means of the Allan deviation *σ_y_*(*τ*) [18]. Allan deviation characterization is commonly used because it allows the determination of the stability of an oscillator in a time interval, τ, for a certain application. The oscillator detection limit, *i.e.*, the smallest frequency deviation that can be detected in presence of noise is equal to [6] *Δf_noise_*(*τ*) *= σ_y_(τ)·f_0_*, where f_0_ is the nominal frequency. In QCM applications, the mass resolution can be obtained by the relationship between the detection limit and Sauerbrey sensitivity [17] of the sensor by Resolution = *Δf_noise_/k*, where $k=2.26\cdot 10^{-6}\cdot f_{o}^{2}$ (Hz g^−1^ cm^2^) is the mass sensitivity coefficient, known as the Sauerbrey coefficient. A detection limit of 2 Hz was calculated by Allan deviation in static conditions (water, without any circulation). Therefore the designed system has a mass resolution of about 357 ng/cm^2^.

**Figure 3.**
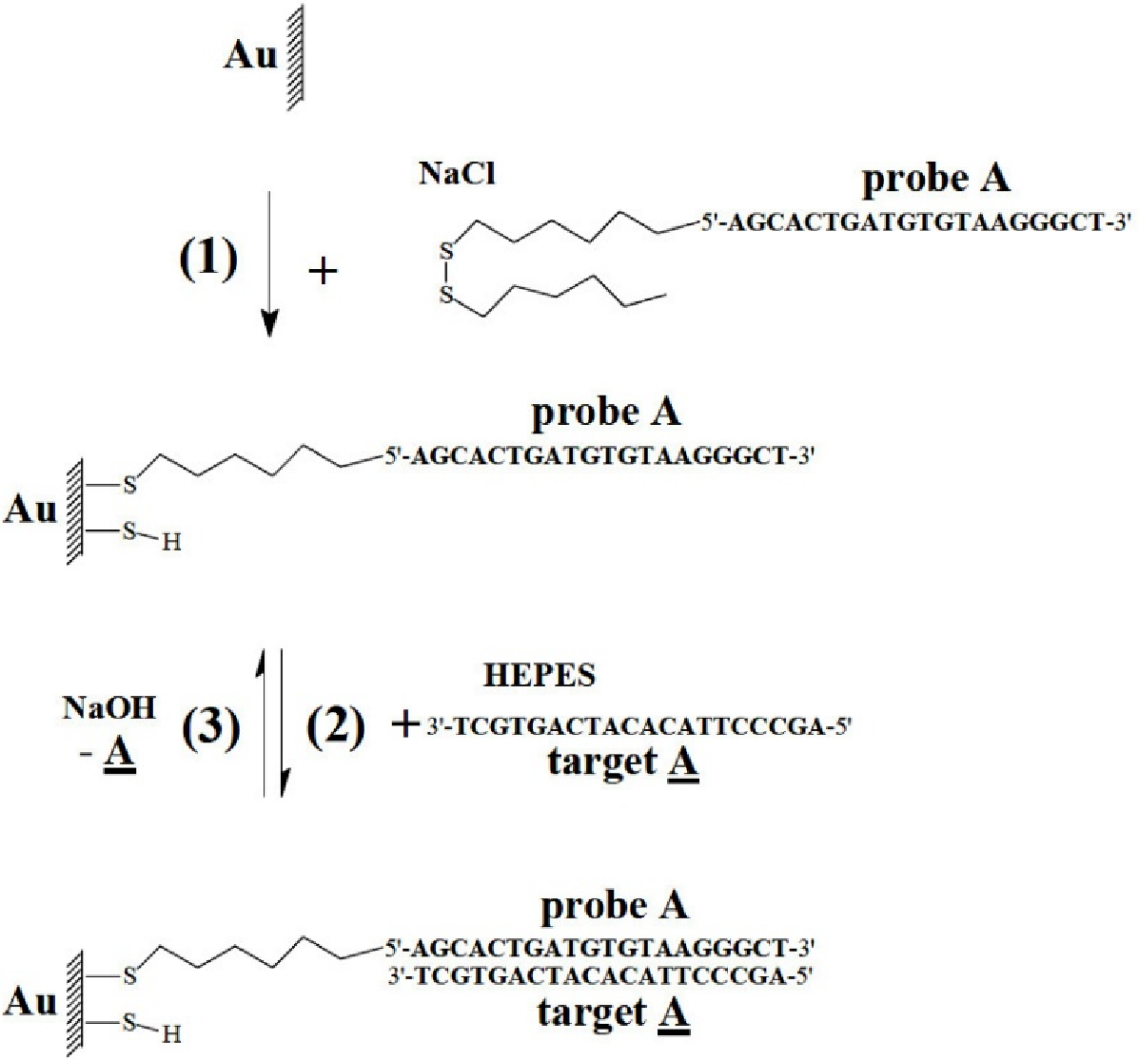
DNA-disulfide biosensor: immobilization of DNA-disulfide probe A (**1**), hybridization of a complementary DNA target A (**2**) and dehybridization of the DNA target A (**3**).

A disulfide-DNA biosensor was designed using the QCM oscillator by immobilization of a 20-base DNA-disulfide probe A in NaCl solution on the gold quartz surface. The immobilization process is illustrated in Figure 3(1). The solution for DNA immobilization was 0.5 M NaCl referred to as “NaCl”. Immobilization of the recognition element on the surface of the transducer, in our case the DNA-disulfide probe A, is a key stage in the construction of a biosensor. The covalent union of the probe DNA with the gold electrode of the quartz takes place thanks to that the used concentration of DNA contains sulfur (S) that will carry out the union between the gold of the electrode and the DNA. To carry out the immobilization the concentration of DNA is added to the NaCl solution and a 50 μL/min constant flow of this solution is maintained in the plexiglass cell in which the quartz resonator is included.

After the immobilization, DNA-disulfide probe A was hybridized in HEPES solution with a complementary DNA target A (2). A/A are 20-base complementary sequences. Hybridization experiments were performed in 0.05 M HEPES, with 0.5 M NaCl, adjusted to pH 7.2 with drops of 1 M NaOH, referred to as “HEPES” [19]. The dehybridization solution was 0.5 M NaOH, with 3 M NaCl, referred to as “NaOH”.

## 3. Results and Discussion

**Figure 4.**
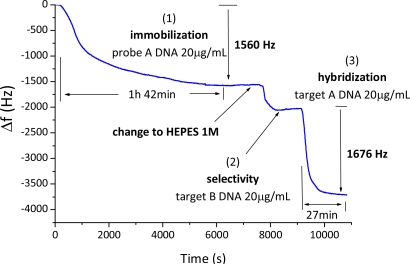
DNA-disulfide biosensor using the designed 50 MHz QCM oscillator circuit: frequency changes during successive circulation of 20 μg/mL DNA-disulfide probe A NaCl solution (**1**), 20 μg/mL DNA target B HEPES solution (**2**), 20 μg/mL DNA target A HEPES solution (**3**).

DNA biosensor oscillator frequency changes recorded during successive circulation of DNA solutions are presented in Figure 4. There is a first Δf_A_ = −1,560 Hz frequency change during circulation of a 20 μg/mL DNA-disulfide NaCl solution attributed to chemical adsorption of the DNA-disulfide probe A on the gold surface of the quartz (1). DNA-disulfide adsorption Δt is equal to 6,120 s. The next frequency shift is attributed to increase of viscosity and density between NaCl and HEPES solutions. There is no frequency shift during circulation of 20 μg/mL non-complementary DNA B HEPES solution indicating that there is no hybridization or non-specific adsorption of the non-complementary DNA strands B (2).

There is a Δf_A_ = −1,676 Hz frequency change during circulation of a 20 μg/mL complementary DNA A solution in HEPES attributed to hybridization of the complementary DNA target A with the biosensor DNA probe A (step 3). The corresponding hybridization ratio η of hybridized DNA strands A, N_A_ *vs.* immobilized DNA-disulfide probes A, N_A_ is estimated to be 58%: η = N_A_/N_A_ = (Δf_A_.M_A_)/(Δf_A_.M_A_), where M_A_ = 6,500 g/mol is the molecular weight of the DNA-disulfide probe and M_A_ = 12,000 g/mol is the molecular weight of the DNA target A. The half-time hybridization reaction Δt calculated as indicated previously is equal to 1,620 s.

The DNA-QCM 50 MHz oscillator biosensor designed is selective, as there is no frequency change of the QCM during circulation of the non complementary DNA B HEPES solution. Also, this 50 MHz DNA biosensor is high sensitive, because there was a −1,676 Hz frequency change during circulation of a 20 μg/mL DNA target A HEPES solution, in front of −55 Hz that can be found using a DNA-QCM 27 MHz oscillator biosensor designed and studied in previous works [5]. With respect to the frequency stability, a detection limit of 2 Hz is calculated by Allan deviation for the best result in static conditions (water, without any circulation). Therefore the designed system has a mass resolution of about 357 ng/cm^2^ in front of the 665 pg/cm^2^ determined for the 27 MHz oscillator in the same conditions [6]. On the other hand, in dynamic conditions the detection limit worsens to 40 Hz with the liquid circulation. Therefore the 50 MHz DNA biosensor has a mass resolution of 7.1 ng/cm^2^. In the case of the 27 MHz oscillator, a detection limit of 20 Hz and a mass resolution of 13.1 ng/cm^2^ are determined in dynamic conditions. Hence, in conclusion, the 50 MHz system improves the mass resolution in static or dynamic conditions (with or without any liquid circulation). Relating to the possible influence of factors as changes of detection buffer or temperature, exactly the same conditions of experiment are used between the 27 MHz and the 50 MHz in term of buffers, temperature, probes and targets. For the buffer changes, the DNA detection is done in the same buffer (HEPES) before and after the addition of the DNA target. So the influence of the buffer is cancelled. For the temperature, all is thermostated, even the electronic part, and AT cut quartz crystals were used. Probe A can also be dehybridized by circulation of a NaOH solution and hybridized again with the complementary DNA target A.

**Figure 5.**
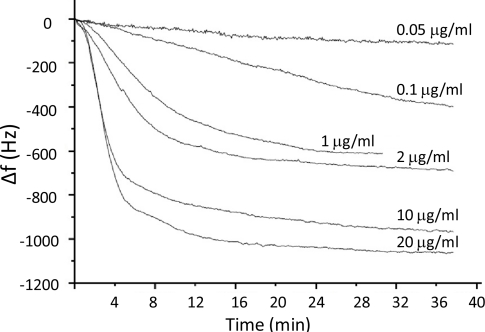
DNA-disulfide biosensor using the 50 MHz QCM oscillator circuit: frequency changes during the circulation of different concentrations of DNA target A HEPES solutions.

Finally, a calibration of the DNA-QCM 50 MHz oscillator biosensor with buffer solutions of different target DNA concentrations was carried out. In Figure 5 the obtained frequency curves are shown. It was found that the designed oscillator is able to detect DNA target A concentrations higher to 50 ng/mL. It can be observed a difference of frequency change during the DNA target detection between Figures 4 and 5. This signal difference is due to that the quartz resonator is not the same in the two figures, and the DNA probe quantity varies from one quartz to another (due to reproducibility of the probe immobilization).

## 4. Conclusions

A high sensitivity DNA biosensor using a QCM electronic oscillator circuit was designed. The oscillator circuitry was adapted to satisfy the Barkhausen condition, even with the quartz immersed in a liquid media and therefore presenting very low quality factors. A study of the frequency noise of the developed QCM system was carried out in order to determine the resolution of the sensor. A mass resolution of 7.1 ng/cm^2^ was founded in dynamic conditions (with liquid circulation). The behavior of the QCM oscillator as DNA biosensor was proved. Results show that the system is able to detect the presence of complementary target DNAs in a solution with high selectivity and sensitivity. DNA target concentrations higher of 50 ng/mL can be detected.

## Figures and Tables

**Table 1.** Experimental values of the BVD equivalent circuit for the quartz in air and immersed in distilled water.

	**R_Q_ (Ω)**	**L_Q_ (μH)**	**C_Q_ (fF)**	**C_P_ (pF)**	**f_S_ (Hz)**	**Q**
**Air**	45	2330	4.34	10.7	50049219.2	16351
**Water**	130	2349	4.34	17.9	49841134.0	5660

## References

[1] Taylor RF, Schultz JS (1996). Handbook of Chemical and Biological Sensors.

[2] Hou X, Jiang L (2009). Learning from nature: Building bio-inspired smart nanochannels. ACS Nano.

[3] Hou X, Guo W, Xia F, Nie FQ, Dong H, Tian Y, Wen LP, Wang L, Cao LX, Yang Y (2009). A biomimetic potassium responsive nanochannel: G-Quadruplex DNA conformational switching in a synthetic nanopore. J. Am. Chem. Soc.

[4] Mannelli I, Minunni M, Tombelli S, Mascini M (2003). Quartz crystal microbalance (QCM) affinity biosensor for genetically modified organism (GMOs) detection. Biosens. Bioelectron.

[5] Lazerges M, Perrot H, Zeghib N, Antoine E, Compere C (2006). *In situ* QCM DNA-biosensor probe modification. Sens. Actuat. B.

[6] Rodriguez-Pardo L, Fariña J, Gabrielli C, Perrot H, Brendel R (2004). Resolution in quartz crystal oscillator circuits for high sensitivity microbalance sensors in damping media. Sens. Actuat. B.

[7] Granstaff VE, Martin SJ (1994). Characterization of a thickness-shear mode quartz resonator with multiple nonpiezoelectric layers. J. Appl. Phys.

[8] Barnes C (1991). Development of quartz crystal oscillators for under-liquid sensing. Sens. Actuat. A.

[9] Sauerbrey G (1959). Verwendung von Schwingquarzen zur Wägung dünner Schichten und zur Mirkowägung. Z. Phys.

[10] Rodríguez-Pardo L, Fariña J, Gabrielli C, Perrot H, Brendel R (2006). Quartz crystal oscillator circuit for high resolution microgravimetric sensors in fluids. Electron. Lett.

[11] Rodríguez-Pardo L, Fariña J, Gabrielli C, Perrot H, Brendel R (2007). Design considerations of Miller oscillators for high sensitivity QCM sensors in damping media. IEEE Trans. Ultrason. Ferroelectr. Freq. Control.

[12] Rodríguez-Pardo L, Fariña J, Gabrielli C, Perrot H, Brendel R (2008). TSM-AW sensors based on Miller XCOs for microgravimetric measurements in liquid media. IEEE Trans. Instrum. Meas.

[13] Matthys RJ (1983). Crystal Oscillators Circuits.

[14] Ehahoun H, Gabrielli C, Keddam M, Perrot H, Cetre Y, Diguet L (2001). EQCM corrosion sensor for solid metals and metal alloys. Application to the dissolution of 304 stainless steel. J. Electrochem. Soc.

[15] Rodríguez-Pardo L, Cao-Paz A, Fariña J, Covelo A, Novoa XR, Pérez C (2010). Water uptake kinetics in anti-corrosion organic films with a high resolution microbalance oscillator sensor. Sens. Actuat. B.

[16] (1995). Wide Bandwith Operational Transconductance Amplifier and Buffer, Texas Instruments.

[17] Bizet K, Gabrielli C, Perrot H, Therasse J (1998). Validation of antibody-based recognition by piezzoelectric transducers through electroacoustic admittance analysis. Biosens. Bioelectron.

[18] Allan DW (1966). Statistics of atomic frequency standards. Proc. IEEE.

[19] Zhou CX, Huang LQ, Li SFY (2001). Microgravimetric DNA sensor based on quartz crystal microbalance: Comparison of oligonucleotide immobilization methods and the application in genetic diagnosis. Biosens. Bioelectron.

